# E-DFu-Net: An efficient deep convolutional neural network models for diabetic foot ulcer classification

**DOI:** 10.17305/bb.2024.11117

**Published:** 2024-09-13

**Authors:** Nouf F Almufadi, Haifa F Alhasson, Shuaa S Alharbi

**Affiliations:** 1 Department of Information Technology, College of Computer, Qassim University, Buraydah, Saudi Arabia

**Keywords:** Diabetic foot ulcers (DFUs), deep learning (DL), image classification, ischemia classification, infection classification, medical imaging processing, medical image, transfer learning

## Abstract

The diabetic foot ulcer (DFU) is a severe complication that affects approximately 33% of diabetes patients globally, often leading to limb amputation if not detected early. This study introduces an automated approach for identifying and classifying DFU using transfer learning. DFU is typically categorized into ischemic and infection states, which are challenging to distinguish visually. We evaluate the effectiveness of pretrained Deep Convolutional Neural Network (DCNN) models for autonomous DFU detection. Seven models are compared: EfficientNetB0, DenseNet121, ResNet101, VGG16, MobileNetV2, InceptionV3, and InceptionResNetV2. Additionally, we propose E-DFu-Net, a novel model derived from existing architectures, designed to mitigate overfitting. Experimental results demonstrate that E-DFu-Net achieves remarkable performance, with 97% accuracy in ischemia classification and 92% in infection classification. This advancement enhances current methodologies and aids practitioners in effectively detecting DFU cases.

## Introduction

Diabetes mellitus is a chronic metabolic disorder characterized by persistent hyperglycemia due to insufficient insulin production or the body’s ineffective use of insulin [1]. Without careful management, this disease can lead to numerous complications, among which diabetic foot ulcers (DFUs) are particularly serious [2]. DFUs affect approximately 34% of diabetic patients during their lifetime, meaning a third will experience this significant complication. Additionally, without proper management and prevention strategies, DFUs can recur. The primary causes of these ulcers are peripheral vascular disease and neuropathy. After the initial onset of a DFU, there is an estimated 40% risk of recurrence within the first year, rising to 60% within three years [3]. This data indicates a high recurrence rate in this population. A recent study by the American Diabetes Association found that over one million individuals with diabetes undergo amputations annually due to their condition [4]. Moreover, DFUs are closely linked to increased cardiovascular disease (CVD) risk and mortality, due to shared risk factors like diabetes, systemic inflammation, and compromised circulation. These factors result in higher mortality rates in individuals with DFUs, often due to cardiovascular complications [5].

**Figure 1.**
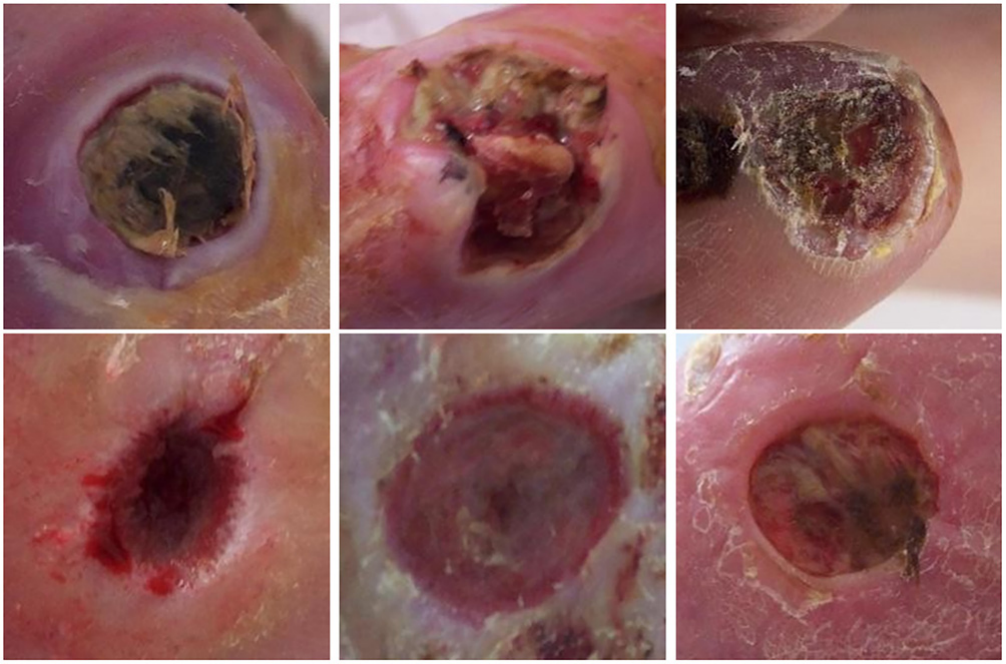
**Samples of DFU Ischemia (first row) and Infection (second row) from DFU-Part(B) dataset [40].** DFU: Diabetic foot ulcer.

Premature identification and appropriate treatment of diabetic foot complications, such as DFUs, are essential in preventing catastrophic outcomes. Health professionals are required to monitor the status of patients surviving DFU to assess the extent of their recovery and recommend appropriate drugs to ward off further complications [6]. DFU presents in two main conditions. The primary condition is ischemia, and the secondary condition is infection, caused by a lack of blood circulation and bacterial sepsis in the wound areas, respectively, as illustrated in Figure 1. Patients with ischemia face a 40% risk of three-year mortality from ischemic gangrene [7]. Additionally, 56% of DFU cases become infected, with 20% risking limb amputation [8–10]. DFUs lower quality of life and increase economic costs. Early detection aids in timely intervention, correct diagnosis, and effective treatment, preventing poor outcomes.

High-quality images are utilized to assess the condition of DFU, playing a crucial role in early diagnosis, monitoring progress, and determining appropriate treatment strategies for each case. This process includes reviewing the patient’s medical history, conducting a thorough examination of the DFU by an expert or podiatrist, and potentially performing additional assessments, such as blood work, physical evaluations, and Doppler studies of the lower extremity blood vessels to develop a treatment plan. However, applying these steps has time limitations [11]. Researchers have been exploring the use of machine learning (ML) and deep learning (DL), along with computer-aided detection systems, to address these limitations in detecting DFU. Deep convolutional neural networks (DCNNs) have shown excellent performance in identifying and categorizing different biological tissues, such as foot skin or brain tumors, due to their ability to generalize various levels of features [12]. It is also important to note that DCNN preprocessing, including techniques, such as data expansion and color normalization, plays an integral role in ensuring accurate and reliable results. The performance of DCNN architectures depends heavily on the quality of the dataset and the training procedure, as these architectures deliver superior results when working with more precise data [13]. Transfer learning can be utilized to enhance performance and address this limitation.

Transfer learning is a valuable methodology that involves gaining knowledge from a broad domain and subsequently applying that knowledge in a confined space, ultimately resulting in a superior classification process compared to what would be achievable if the domain were trained from the beginning [14]. Transfer learning encompasses two main approaches: modifying the convolutional network or maintaining the static nature of its layers. Using a pre-existing model for fine-tuning is sufficient for classification tasks, rather than building a DCNN model from scratch. Furthermore, using a pretrained DCNN model as a basis for transfer learning offers several advantages [15]. Firstly, pretrained CNN models have already been trained on large datasets, allowing them to learn rich and discriminative features. Secondly, pretrained models save time and computational resources since they do not require training on a large dataset. Colors and edges are extracted by the lower layers of the pretrained model, while objects and contours are captured by the upper layers [16]. Therefore, using the expertise developed from a pretrained DCNN model in another area, such as identifying brain tumor types in MRI images, we can enhance classification accuracy by extracting significant features.

Ischemia and infection can be assessed using ML and DL techniques based on the visual appearance of DFU images. For ischemia, tissue death in the foot, which appears as black gangrenous toes, indicates ischemia, while for infection, purulent discharge and redness in and around the ulcer indicate infection [17]. Healing progress assessment and the prevention of amputation depend on accurate identification of both infection and ischemia. The main contributions of this paper are as follows:

- Introducing a method for automatic DFU identification using DCNN transfer learning. This involves testing various pretrained DCNN models (e.g., InceptionV3 and EfficientNetB0) for binary classification of infections and ischemia in DFUs, utilizing the DFU-Part (B) dataset for effective feature extraction [10].
- To address the problems of overfitting and the disappearing gradient, we suggest a combination of the Proposed Head Model and the pretrained DCNN model, enhancing the overall result.

The rest of this paper follows the following structure: Section 2 presents related works on DFU binary classification methods for ischemia and infection. Section 3 describes the methodology used to develop the proposed framework, while Section 4 discusses the dataset used. Section 5 includes the experimental evaluation and discussion of the results. Section 6 concludes the paper.

### Diabetic foot binary classification methods for ischemia/infection

Goyal et al. [18] introduced innovative DL approaches for detecting DFU in real time. These methods involve several stages, including using a CNN as a feature extractor, generating proposals and refining them, and finally employing a RoI classifier along with a bounding box regressor. Subsequently, Cassidy et al. [19] used four distinct models–R-FCN, faster R-CNN, ResNet-101, and YOLOv5–to identify DFU. Ibrahim and Abdulazeez [20] used a method that involved several sets of pretrained models, such as Inception-V3, InceptionResNetV2, and ResNet50, as part of an ensemble learning approach. This approach consists of using multiple models to achieve better predictive performance compared to predictions that could be obtained from any of the individual models. The models were further subjected to support vector machines (SVMs) to produce valuable predictions. A new data augmentation method was implemented to apply to the region of the images after extracting these deep features. Amin et al. [21], on the other hand, introduced a 16-layer CNN model. By using deep features from CNN, they traded off, classifying and obtained significant accuracy in diagnosing foot wounds.

The CNN-based strategy of Al-Garaawi et al. [22] involves the extraction of texture information from RGB DFU images, which is then used as input as input for the CNN model. Initially, their approach includes texture data extracted at the first stage of the model; then it uses the texture image to classify DFUs. The created CNN achieves a 99% accuracy rate for recognizing ischemia and a 74% success rate for identifying infection.

Al-Garaawi et al. [24] proposed a three-phase system including feature selection, feature fusion, and DFU classification. In the main body feature picking stage, such textural features as HOG, Gabor, and deep features are selected with a GoogLeNet CNN model. Next, these selected features are aggregated into a single vector. The aggregate vectors are then sent to the random forest algorithm for classifying DFUs. The accuracy of the experiment turned out very high for the classification of ischemia and 132 infections.

**Table 1:** Studies on binary classification for infection and ischemia

**Author [ref.]**	**Year**	**Model**	**Dataset**	**Evaluation criteria**	**Result**
Goyal et al., [11]	2020	Ensemble CNN (Inception-V3, Inception ResNetV2, and ResNet50)	DFU-Part(B) [11]	Accuracy	Ischemia: 90%, infection: 73%
Amin et al., [21]	2020	Proposed CNN	DFU-Part(B) [11]	Accuracy	Ischemia: 97.9%, infection: 99.6%
Al-Garaawi et al., [22]	2022	CNN DFU-RGB-T EXT-NET	DFU-Part(A) [23] and DFU-Part(B) [11]	Accuracy	Ischemia: 99%, infection: 74%
Al-Garaawi et al., [24]	2022	GoogLNet CNN with RF	DFU-Part(B) [11]	Accuracy	Ischemia: 92%, infection: 73%
Xu et al., [25]	2022	Transformer based DeiT model with class knowledge banks (CKBs)	DFU-Part(B) [11]	Accuracy	Ischemia: 90.9%, infection: 78%
Das et al., [26]	2022	ResKNet	DFU-Part(B) [11]	Accuracy	Res4Net for ischemia: 97.8% Res7Net for infection: 80%
Toofanee et al., [27]	2023	DFU-SIAM	DFU-Part(B) [11]	Macro-F1 score, F1 score	0.623, ischemia: 0.549, infection: 0.628
Das et al., [28]	2024	HCNNet	DFU-Part(B) [11]	AUC	Ischemia: 0.999

AUC: Area under the curve; DFU: Diabetic foot ulcer.

Xu et al. [25] introduced a novel method for utilizing class knowledge banks to harvest and stockpile class knowledge from datasets, an approach used in image prediction. The literature has reported the development of DL models that have demonstrated significant efficacy in the automated classification of DFUs, achieving classification accuracies approaching 78% for the identification of infection and an impressive 90.9% for the recognition of ischemia using the same imaging data as input. This indicates that DL techniques have been highly successful in addressing these clinically relevant classification problems and hold promise for practical application in the diagnosis and management of diabetic foot complications. Das et al. [26] introduce a revolutionary CNN-based approach for the separation of ischemia from infection. Despite the different layers and network types used, the achieved accuracy was very high, reaching about 97.8%, but only for ischemia recognition, using the enhanced Res7 extension network.

Looking at the accurate classification of DFUs, Toofanee et al. [27] introduced a new deep neural network (DNN) approach embedded with ML, composed of a siamese neural network (SNN) and vision image transformer (ViT). Their technique proficiently categorized four classes of DFUs, including None, Ischemia, Both, and Infection. Simultaneously, Das et al. [28] introduced a Hybridized CNN (HCNNet) by incorporating multiple hybridized blocks, such as inception, residual, dense, and squeeze-and-excite (SE) blocks. This designed technique is potentially proficient as it can adjust various optimizers and learning rates during the training of the model (Table 1).

## Materials and methods

### The proposed E-DFu-Net framework

**Figure 2.**
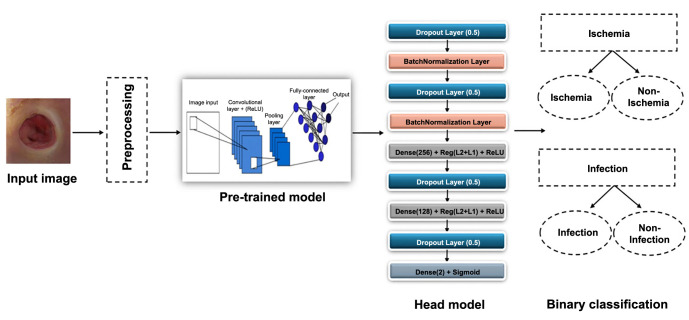
The framework for the E-DFu-Net classification of infection and ischemia.

This study aims to explore the performance of different CNN-pretrained models with the proposed head model, which is placed at the end of the CNN-pretrained models to overcome the overfitting problem in both ischemia classification and infection classification. A T4 GPU accelerator was used with Python in the Google Colab Pro version of the application. Various libraries, including Sklearn, Pandas, Numpy, glob, and Matplotlib, have been used to implement the work using the TensorFlow platform and Keras framework. Figure 2 illustrates the framework of our proposed model.

### Dataset

**Figure 3.**
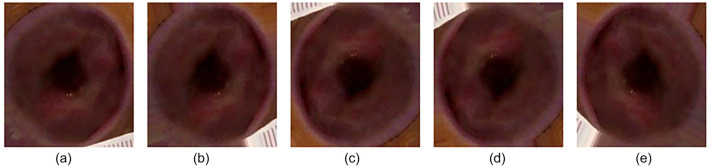
A sample of Ischemia images: (A) before augmentation; (B–E) newly generated Ischemia images after augmentation.

**Figure 4.**
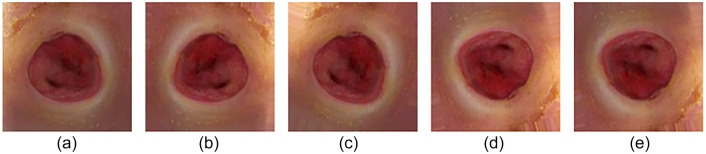
A sample of infection images: (A) before augmentation; (B–E) newly generated infection images after augmentation.

The DFU-Part (B) [11] dataset was used for the evaluation and validation of the proposed model. This dataset is used for the binary classification of ischemia and infection in DFU. A total of 1249 images of ischemia are included in the dataset, and 210 images of non-ischemia are also included. There are also 628 images of infection and 831 images of non-infection. The authors of the DFU-Part (B) dataset applied a natural data augmentation technique to make the dataset balanced. In order to identify ulcers in the region of interest (ROI), data augmentation is proposed. The data augmentation technique they proposed is more appropriate for DFU assessment compared to common data augmentation methods, due to the risk of missing the ROI in DFU images when using techniques such as cropping, translation, and random scaling. The authors generated 9870 augmented images through natural augmentation, with 4935 images associated with ischemia and 4935 with non-ischemia, following the selection of the chosen dataset. Approximately 4890 augmented images were generated, including 2945 related to infection and 2945 images related to non-infection. Table 2 shows the total number of original and augmented images in each class of the dataset. The size of the images in the dataset varies between 1600 × 200 and 3648 × 2736 pixels. Therefore, the images were resized to 224 × 224 pixels in the preprocessing phase to reduce the training time. Due to the better performance of DL models with larger datasets [29], data augmentation was applied in this study to increase the number of images. Samples of both ischemia and infection images before and after the data augmentation process are shown in Figures 3 and 4, respectively.

**Table 2:** Summary of the number of images in the DFU-Part (B) dataset for each class before and after natural data augmentation and after our augmentation process

**Classification type**	**Class**	**No of images**	**No of natural augmented images**	**No of natural augmented images (Ours)**
Ischemia Classification	Ischemia, Non-ischemia	1249 210	4935 4935	6062 6062
Infection Classification	Infection Non-infection	628 831	2945 2945	4212 4212

DFU: Diabetic foot ulcer.

The data augmentation techniques used include rotation, horizontal flip, and vertical flip. Other methods such as cropping, translation, and random scaling were excluded to prevent the risk of overlooking crucial parts in the DFU images. Additionally, both online and offline data augmentation methods were tested to determine which provided better results. We found that offline data augmentation, where the augmented images are created and saved for later use in training, provided better results than applying data augmentation online, where images are augmented during the training of the model. The ischemic dataset consists of 12,124 images (ischemia and non-ischemia), an increase of 9870 from its previous number. Similarly, the infection dataset now includes 8424 images (infection and non-infection), an increase from the original number of 4890 images. The datasets of ischemia and infection were further divided into training, testing, and validation sets in the respective ratios of 70%, 20%, and 10%.

Further experiments, involving different ratios to divide the dataset, were performed. After conducting multiple experiments, it was decided to allocate 70% of the dataset for training, 20% for validation, and 10% for testing. The specified ratio resulted in the best performance compared to other evaluated ratios.

### Experimental evaluation

We utilized several pretrained CNN models to evaluate various CNN architectures for classifying infection and ischemia images obtained from a selected dataset. We aimed to achieve the binary classification of each condition and pinpoint the best-performing CNN model for labeling infection and ischemia. We initially assessed the individual performance of these CNN models before introducing the proposed head model to enhance their efficiency. The models used were EfficientNetB0 [30], DenseNet121 [31], ResNet101 [32], VGG16 [33], InceptionV3 [34], MobilenetV2 [35], and InceptionResnetV2 [36]. The CNN models were previously trained to leverage the ImageNet dataset for transfer learning. The CNN models were tested using diverse hyperparameter values to ensure the best possible results in comparison to other hyperparameter values across all models. Table 3 illustrates the various hyperparameter values employed by the models.

**Table 3:** Hyperparameter values

**Parameter name**	**Value**
Optimizer	Adamax
Learning rate	0.001
Patience of the EarlyStopping	20
Batch size	32
Epochs	100

### Proposed head model

**Figure 5.**
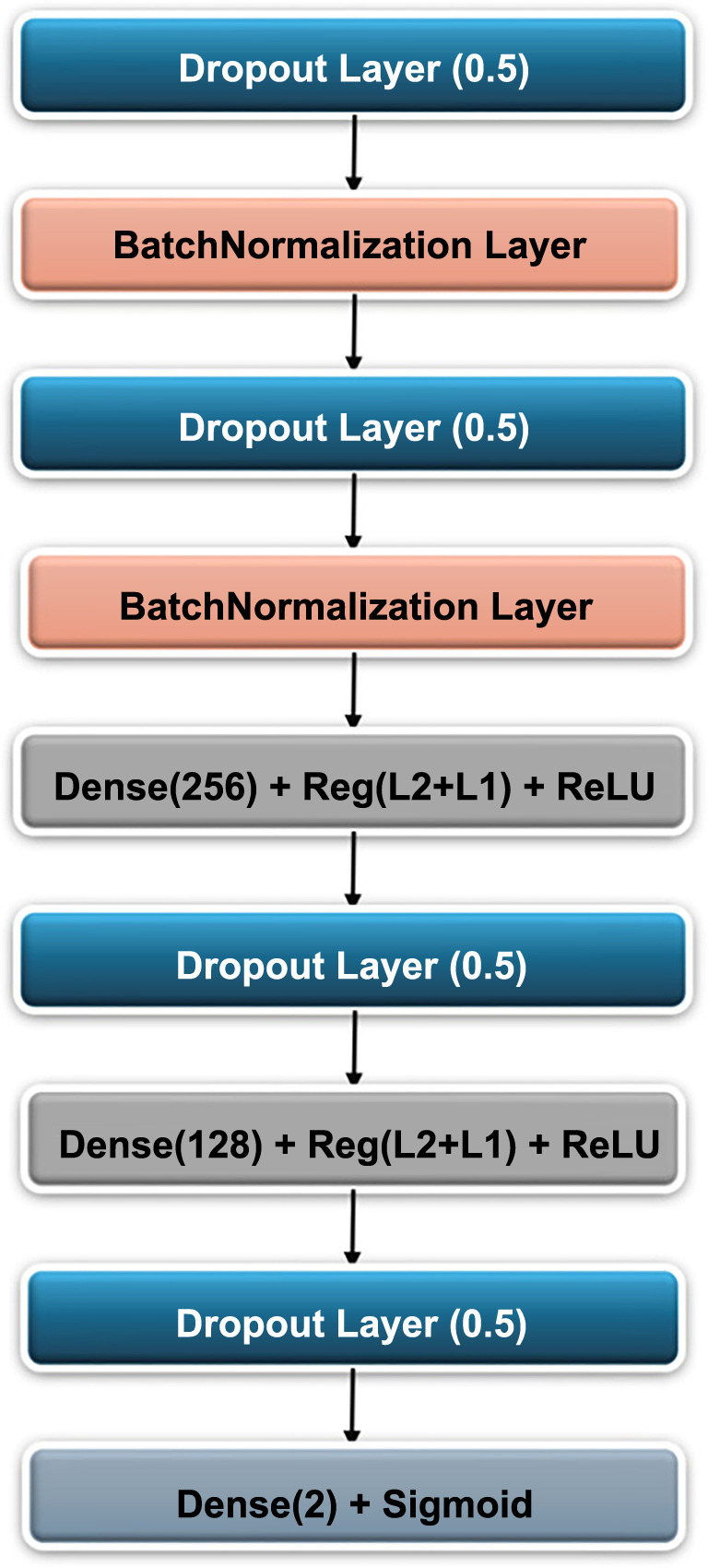
Illustrated head model structure with optimized layers for efficient data processing and enhanced learning accuracy.

A supplementary head was introduced to tackle overfitting in the pretrained CNN models. The idea emerged when we analyzed the loss graphs of the pretrained models and observed that as the model was trained, the training loss decreased continuously, while the validation loss had a different curve. In the beginning, the validation loss dropped, but after a certain stage, it started to rise, indicating overfitting. This finding led us to develop a novel head model, which comes after the CNN models, to combat overfitting and enhance performance. We refined the structure of the head model through several experiments where we used different layer types, different numbers of layers, and various layer combinations to find the most effective design that can save CNN models from overfitting. Figure 5 shows the design of the suggested head model.

The resulting model showed significantly better-fitting learning curves in the loss graphs, indicating that the losses during both training and validation decreased more than in the cases without the head. The impact of this head model is evident in improving learning outcomes and mitigating overfitting, as both training and validation losses decrease noticeably. The head model consists of multiple layers placed together in a specific order, addressing the problem of overfitting and improving the ultimate performance of the models. Table 4 indicates that incorporating multiple layers in a specific sequence effectively addressed overfitting issues and enhanced performance outcomes.

We employed different pretrained CNN models to evaluate their ability to classify images of infection and ischemia. This phase involved independently assessing the performance of each model before integrating the proposed head model and using the outputs with ML classifiers. The models included DenseNet121, ResNet101, InceptionV3, InceptionResNetV2, VGG16, EfficientNetB0, and MobileNetV2. These CNN models were pretrained using transfer learning, allowing them to acquire rich and distinctive features, thereby reducing the time and computing resources needed by transferring knowledge from a large-scale domain to a specific task. Transfer learning was implemented on several CNN models using the ImageNet dataset. The early layers of the pretrained models capture fundamental features like colors and edges, while the deeper layers recognize more complex features such as objects and contours. This approach leverages the models’ ability to extract important features, improving classification accuracy for identifying DFU types from images. The performance of the CNN models is greatly influenced by the training process. For transfer learning, we maintained the original architectures, removed the top layers, and applied ImageNet for initial weights. This excluded the final dense layer responsible for converting the 1280-dimensional feature vector into predictions for the 1000 ImageNet categories. This modification preserves the convolutional base of the models, which generates feature maps, making it especially beneficial for transfer learning. Additionally, we set specific layers to layer.trainable ═ False to prevent their weights from changing during training.

A dropout layer was implemented to reduce overfitting and enhance the CNN model’s generalization capabilities. The dropout layer randomly sets a fraction of neurons in a layer to be inactive, thus reducing the number of parameters in the model and preventing overfitting. This helps the model generalize better to unseen data. By using a dropout rate of 0.5, 50% of the input neurons are randomly deactivated during each training iteration, which helps reduce model complexity and prevents reliance on specific neurons. This stochastic process encourages the network to learn more robust features. Additionally, a BatchNormalization layer was used, which normalizes the inputs of each layer. This not only speeds up training but also stabilizes the learning process.

After conducting several experiments, a selection of dropout layers and batch normalization layers was chosen and organized to utilize the configuration that resulted in optimal outcomes. The number of dense layers and their respective units was investigated in depth to attain the highest level of productivity. Moreover, a combination of activation techniques, such as kernel regularizer, L1 and L2 regularization, activity regularizer, and bias regularizer, was used to prevent overfitting and control CNN model complexity. These activation strategies were chosen through multilevel experimentation to achieve the appropriate settings for engaging them. Using the ReLU activation function in dense layers, the final layer of this model includes a dense layer with two units, enabling binary classification for infection (infection or non-infection). The same approach applies to ischemia classification (ischemia or non-ischemia). Furthermore, the last layer utilizes the sigmoid activation function, which is ideal for binary classification.

### Evaluation parameters

The proposed approach produces a binary classification, applied to categorize images as either infection or non-infection, and similarly for ischemia images as ischemia or non-ischemia. Various assessment metrics are used to evaluate classification results. These computations depend on true-positive (TP) and true-negative (TN) values, which represent correctly identified cases, and false-positive (FP) and false-negative (FN) values, which indicate misclassified cases [37].

**Table 4:** The details of the proposed head model structure

**Layer name**	**Values**
Dropout layer	0.5 Rate
BatchNormalization	Default values
Dropout layer	0.5 Rate
BatchNormalization	Default values
Dense layer	256 Units + kernel reg ═ l2 + activity reg ═ l1 + bias reg ═ l1 + ReLU
Dropout layer	0.5 Rate
Dense layer	128 Units + kernel reg ═ l2 + activity reg ═ l1 + bias reg ═ l1 + ReLU
Dropout layer	0.45 Rate
Dense layer	2 Units + Sigmoid


(1)

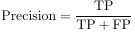





(2)

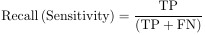





(3)

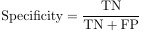





(4)

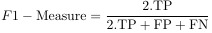





(5)

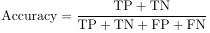




Additionally, the Area under the curve (AUC) measures how well the classifier can distinguish between two classes.


(6)

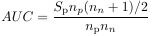




*S*_*p*_ is the sum of all positive example. *n*_*p*_ is the number of positive examples. *n*_*n*_ is the number of negative examples.

## Results

### Visualization of loss and accuracy curve

**Figure 6.**
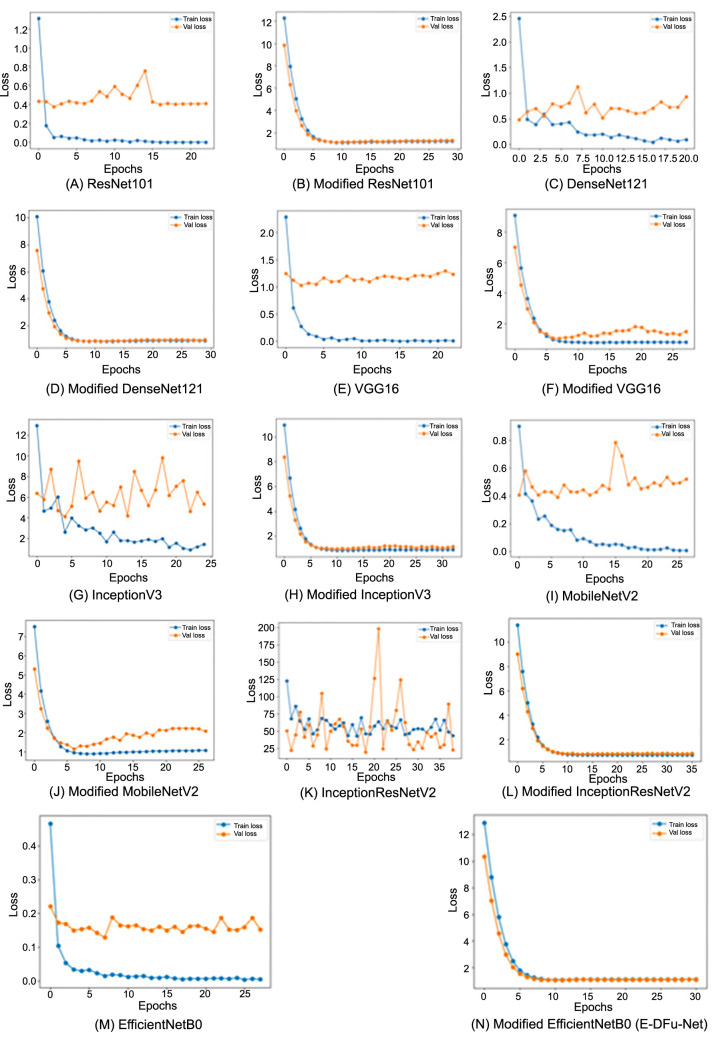
The training and validation loss curves for each pretrained model, shown before and after incorporating the proposed head model, illustrate improvements in ischemia classification performance.

**Figure 7.**
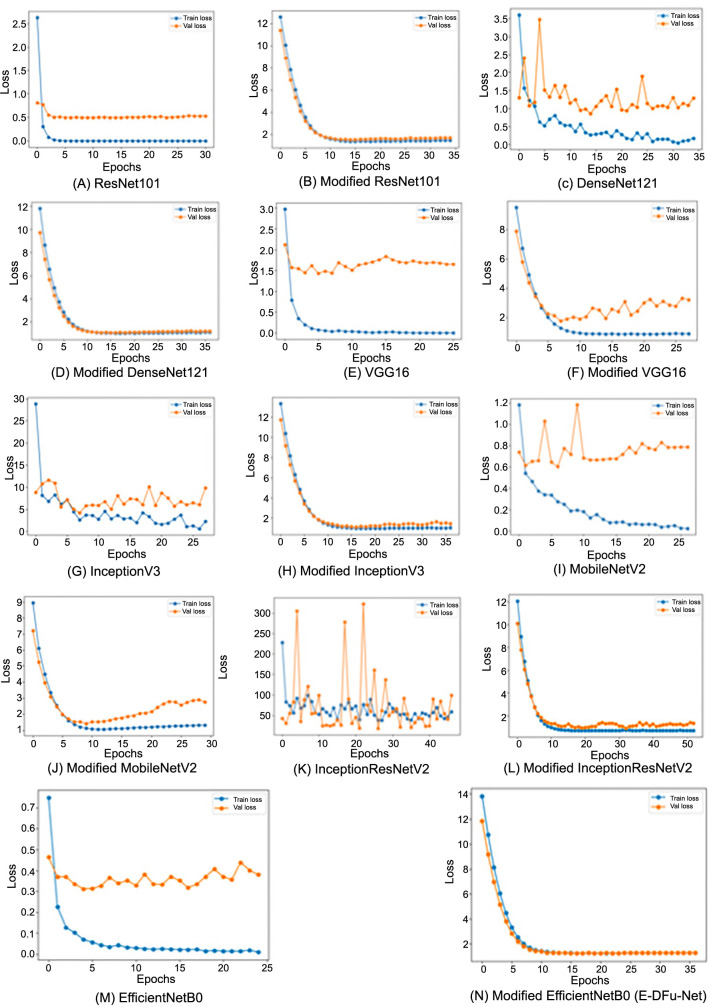
The training and validation loss curves for each pretrained model, displayed before and after incorporating the proposed head model, demonstrate enhanced performance in infection classification.

**Figure 8.**
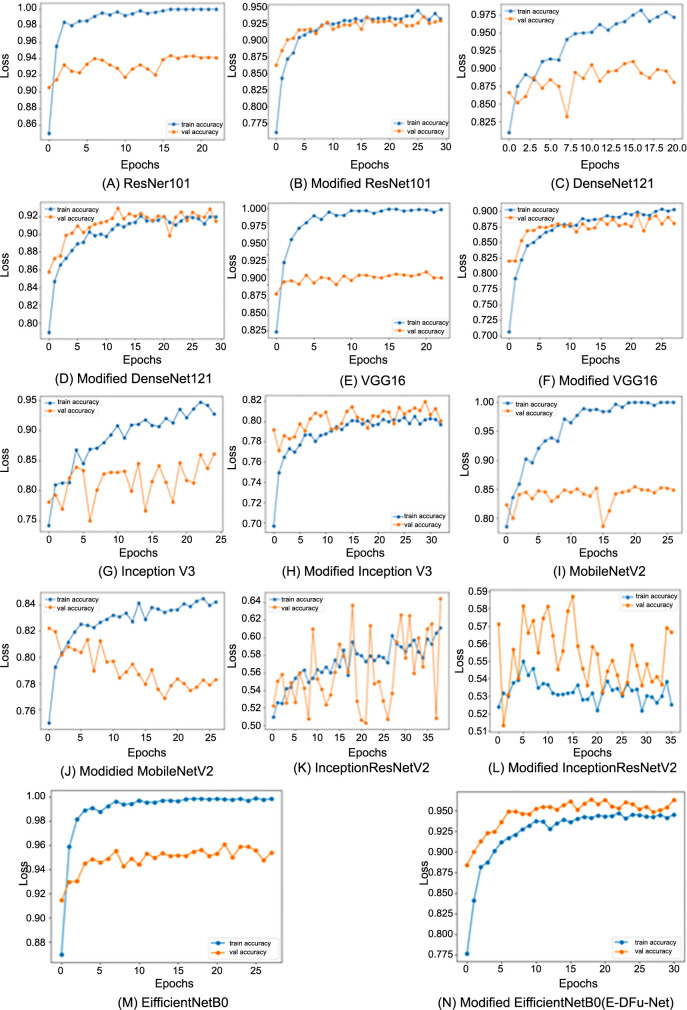
The training and validation accuracy curves for each pretrained model, shown before and after integrating the proposed head model, reveal improved performance in ischemia classification accuracy.

**Figure 9.**
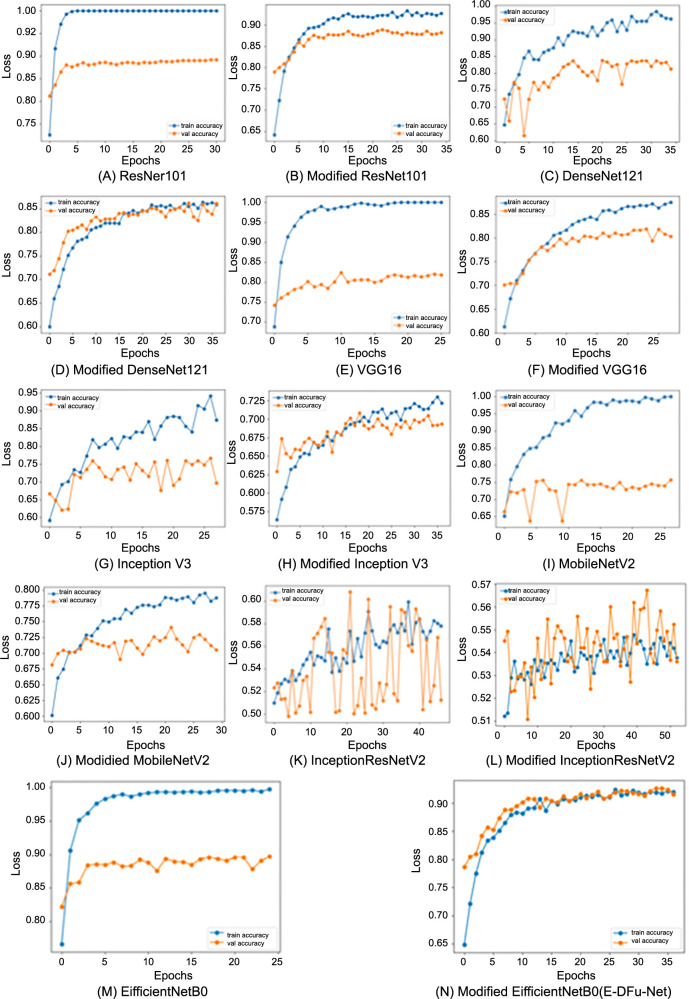
The training and validation accuracy curves for each pretrained model, displayed before and after integrating the proposed head model, demonstrate significant improvements in infection classification accuracy.

The loss curves displaying the classification of ischemia and infection before and after the CNN models were integrated with the suggested head models are presented in Figures 6 and 7, respectively. These graphs clearly show that the CNN models achieve optimal learning with the designed head models in place. Before adding the head models, the loss graph indicated overfitting issues, as the training loss consistently decreased while the validation loss initially dropped and then increased. Once the head models were integrated, both training and validation curves declined, indicating improved learning. The CNN model’s accuracy trend is displayed in Figure 8. The model’s accuracy trend for ischemia classification is visible both before and after adding the head model. The second figure, Figure 9, shows the accuracy trend for infection classification. The images visually indicate the difference in validation accuracy before and after adding the head model. In addition, the integration of the proposed head model decreased the gap in training and validation accuracy, resulting in improved model accuracy overall.

### Comparative analysis

We present the results of the evaluation of the CNN models on the ischemia and infection classification tasks and inspect the performance of DenseNet121, ResNet101, InceptionV3, InceptionResNetV2, VGG16, EfficientNetB0, and MobileNetV2, while using pretrained weights from the ImageNet dataset. Table 5 displays the ischemia results, and Table 6 depicts the results of the infection task. The EfficientNetB0 model achieved the best scores and outperformed the competition. The results in Tables 5 and 6 indicate that the EfficientNetB0 and ResNet101 models performed exceptionally well on the DFU-Part (B) dataset for classification tasks, showing high accuracy in identifying both ischemia and infection. Although the ResNet101 model trained faster, with a difference of less than three minutes, the superior performance of EfficientNetB0 makes it a preferable choice. Additionally, DenseNet121 and VGG16 also achieved strong and similar metrics in DFU binary classifications, with DenseNet121 requiring less training time than VGG16. Overall, EfficientNetB0, ResNet101, and DenseNet121 are effective DL models for DFU binary ischemia and infection classifications.

**Table 5:** Ischemia classification results of CNN pretrained models

**Name of pre-trained CNN model**	**Acc.**	**Prec.**	**Sens.**	**Sp.**	**F1 score**	**AUC**	**Time (s)**
EfficientNetB0 [30]	**0.947**	**0.950**	**0.943**	**0.950**	**0.947**	**0.947**	1895
ResNet101 [32]	0.925	0.912	0.940	0.909	0.926	0.925	1688
DenseNet121 [31]	0.899	0.883	0.920	0.878	0.901	0.899	1135
VGG16 [33]	0.886	0.892	0.878	0.894	0.885	0.886	1632
InceptionV3 [34]	0.819	0.766	0.919	0.719	0.835	0.819	1105
MobileNetV2 [35]	0.832	0.809	0.869	0.795	0.838	0.832	880
InceptionResNetV2 [36]	0.560	0.534	0.950	0.21	0.684	0.560	2163

**Table 6:** Infection classification results of CNN pretrained models

**Name of pre-trained CNN model**	**Acc.**	**Prec.**	**Sens.**	**Sp.**	**F1 score**	**AUC**	**Time (s)**
EfficientNetB0 [30]	**0.904**	**0.886**	**0.926**	**0.881**	**0.906**	**0.904**	831
ResNet101 [32]	0.896	0.917	0.872	0.921	0.894	0.896	2109
DenseNet121 [31]	0.829	0.814	0.853	0.805	0.833	0.829	653
VGG16 [33]	0.827	0.822	0.834	0.819	0.828	0.827	1010
InceptionV3 [34]	0.763	0.824	0.668	0.857	0.738	0.763	725
MobileNetV2 [35]	0.747	0.717	0.817	0.677	0.764	0.747	747
InceptionResNetV2 [36]	0.535	0.522	0.810	0.260	0.635	0.535	1193

According to the data presented in Tables 7 and 8, the modified EfficientNetB0 and ResNet101 models exhibited superior performance in classifying the DFU-Part (B) dataset. Both models achieved high accuracy in identifying ischemia and infection cases. Notably, the training duration for the EfficientNetB0 model was substantially reduced compared to that of the ResNet101 model. Furthermore, the modified DenseNet121 and VGG16 models also performed well, with metrics that were closely matched, although DenseNet121 required less training time than VGG16. Conversely, the MobileNetV2 model, both before and after modification, demonstrated the shortest computational time but had the lowest accuracy. Overall, the findings indicate that the modified EfficientNetB0 model outperformed other transfer learning models in the binary classification of ischemia and infection in DFU cases.

**Table 7:** Ischemia classification results of CNN pretrained models with the proposed head model

**Name of the modified model**	**Acc.**	**Prec.**	**Sens.**	**Sp.**	**F1 score**	**AUC**	**Time (s)**
Modified EfficientNetB0 (E-DFu-Net)	**0.965**	**0.959**	**0.971**	**0.958**	**0.965**	**0.965**	1263
Modified ResNet101	0.933	0.939	0.925	0.940	0.932	0.933	2383
Modified DenseNet121	0.902	0.922	0.879	0.925	0.900	0.902	1226
Modified VGG16	0.878	0.897	0.853	0.902	0.875	0.878	1581
Modified InceptionV3	0.775	0.854	0.665	0.886	0.748	0.775	1173
Modified MobileNetV2	0.785	0.766	0.818	0.751	0.792	0.785	1046
Modified InceptionResNetV2	0.638	0.596	0.851	0.425	0.701	0.638	2540

**Table 8:** Infection classification results of CNN pretrained models with the proposed head model

**Name of the modified model**	**Acc.**	**Prec.**	**Sens.**	**Sp.**	**F1 score**	**AUC**	**Time (s)**
Modified EfficientNetB0 (E-DFu-Net)	**0.919**	**0.951**	**0.883**	**0.954**	**0.916**	**0.919**	1209
Modified ResNet101	0.899	0.918	0.876	0.921	0.896	0.899	2311
Modified DenseNet121	0.868	0.935	0.791	0.945	0.857	0.868	981
Modified VGG16	0.847	0.915	0.765	0.928	0.833	0.847	1143
Modified InceptionV3	0.707	0.761	0.604	0.810	0.673	0.707	1241
Modified MobileNetV2	0.738	0.741	0.732	0.744	0.736	0.738	841
Modified InceptionResNetV2	0.561	0.732	0.194	0.928	0.307	0.561	2740

**Figure 10.**
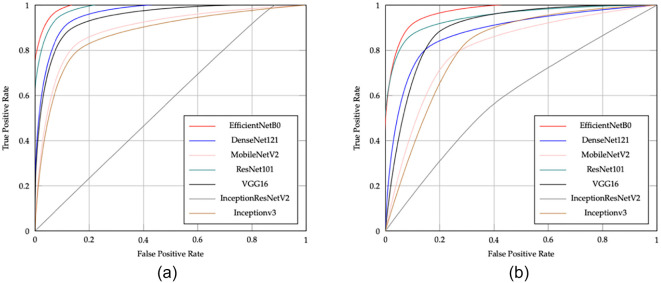
The ROC curves of the CNN pretrained models are presented for (A) ischemia and (B) infection classification, highlighting their diagnostic performance and ability to distinguish between conditions in each task.

Figure 10A shows the ROC curves for the CNN models in ischemia classification, while Figure 10B illustrates the ROC curves for the CNN models in infection classification. The EfficientNetB0 CNN model shows excellent performance in classifying both ischemia and infection, as indicated by its ROC curve being closer to the top left corner. This suggests that EfficientNetB0 has a higher TP rate and a lower FP rate than other models, making it an ideal choice for applications that require high accuracy, such as medical diagnosis.

**Figure 11.**
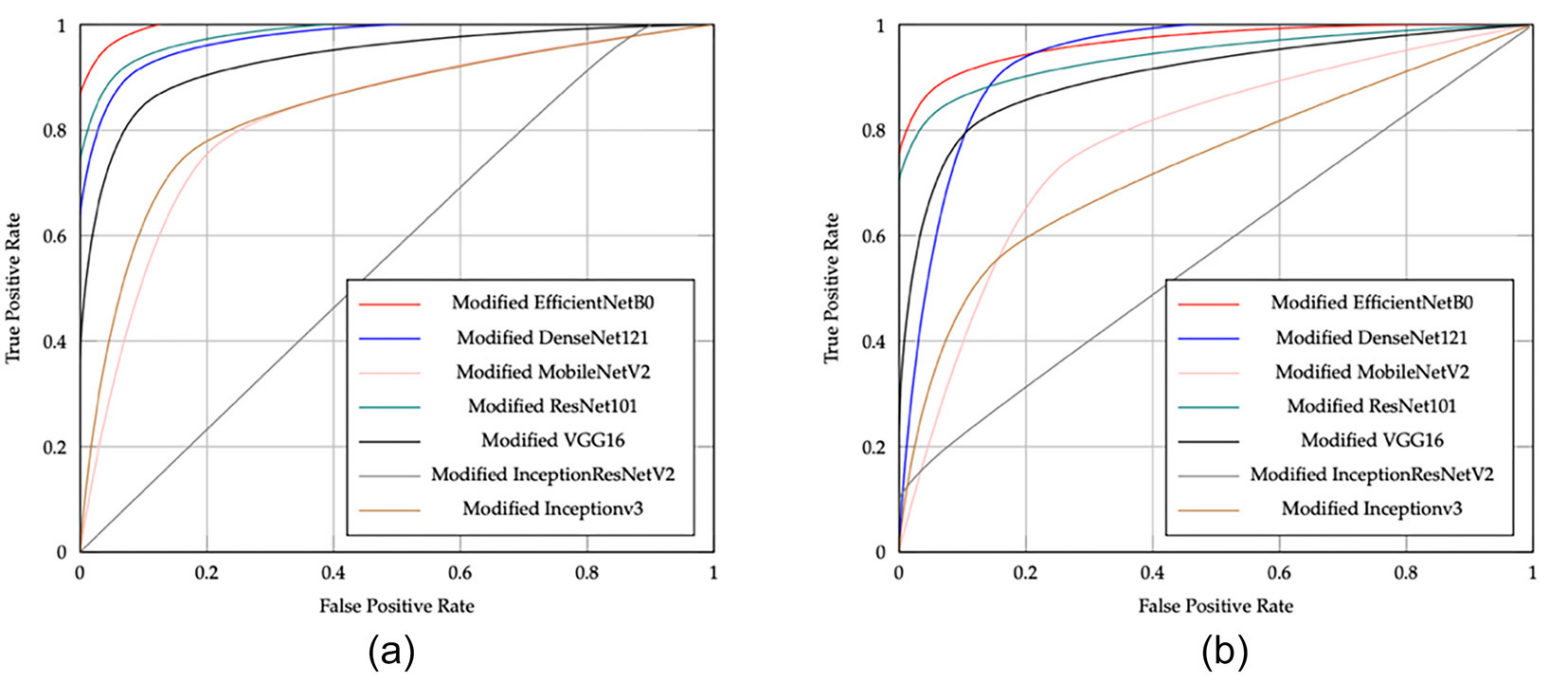
The ROC curves of the CNN pretrained models with the proposed head model are shown for (A) ischemia and (B) infection classification, illustrating enhanced diagnostic performance and discrimination ability in both tasks.

**Figure 12.**
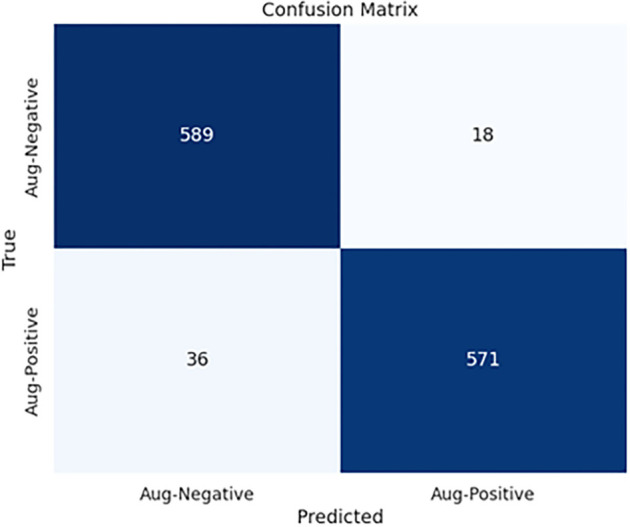
Confusion matrix of EfficientNetB0 with the proposed head in ischemia classification.

**Figure 13.**
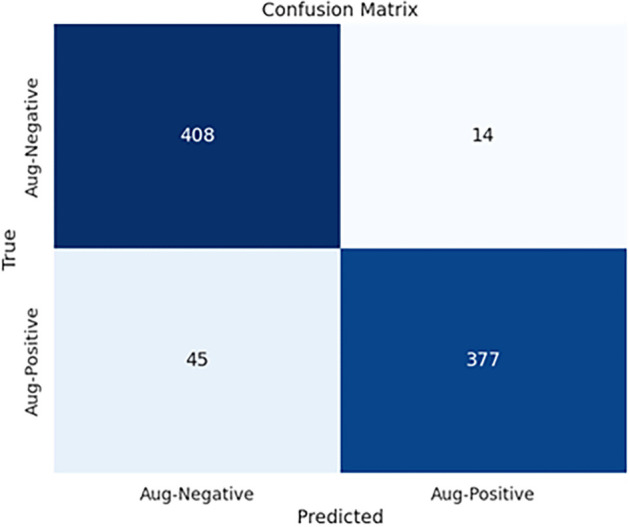
Confusion matrix of EfficientNetB0 with the proposed head model in infection classification.

### Analysis of modified architecture

In Table 7, the findings of the CNN models that utilize the suggested head model for categorizing ischemia are exhibited. Additionally, Table 8 illustrates the outcomes of the models that employed the suggested head model for detecting infection. The findings highlight the superior performance of combining the head model with the EfficientNetB0 model in classifying both ischemia and infection compared to other models using the same methodology. Notably, this combination managed to outperform its generic counterparts and achieve the most impressive scores in differentiating the conditions. Specifically, for the detection of ischemia, the combination of the EfficientNetB0 model and the suggested head model yielded an accuracy of 0.965, a sensitivity of 0.971, and a precision of 0.959. As for infection identification, the same combination secured an accuracy of 0.919, a sensitivity of 0.883, and a precision of 0.951.

After incorporating the suggested head model into the CNN models, a significant improvement in ischemia classification was observed for EfficientNetB0, ResNet101, DenseNet121, and InceptionResNetV2. Furthermore, the integration of the proposed head model yielded superior performance in the classification of infections for ResNet101, DenseNet121, EfficientNetB0, InceptionResNetV2, and VGG16. In summary, the inclusion of the suggested head model contributed to the overall improvement in the accuracy of all CNN models by mitigating the tendency to overfit, which is frequently encountered in these types of models. By including the head model described in this study, we were able to improve the performance of our CNN models. This is reflected in the fluctuations in the loss and accuracy graphs in Figures 6–9 throughout training for both classifying whether an image is symptomatic of ischemia and determining if a COVID-19-infected person has pneumonia, as well as whether the pneumonia is viral or bacterial.

Possessing models that showcase an ideal learning curve is more crucial than having models with superior precision that are susceptible to overfitting. By incorporating the recommended head model, the learning curve is significantly improved, successfully addressing the overfitting issue. Thus, the models convey superior generalizability and precision because the head model fits the appropriate complex structure, making them more reliable for real-life utility.

In Tables 7 and 8, the temporal metric shows a slight increase following the inclusion of the head model in the majority of the models. This is due to an escalation in complexity brought about by the added layers and regularization measures, such as dropout, L1, and L2, which have been implemented to combat overfitting and enhance generalizability to new and unseen data points.

**Figure 14.**
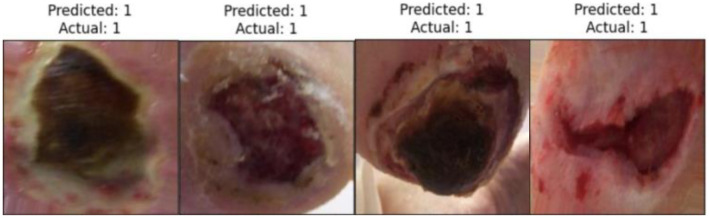
A sample of correctly classified ischemia images in the ischemia classification task using E-DFu-Net, highlighting its precision and efficacy in identifying ischemic conditions.

**Figure 15.**
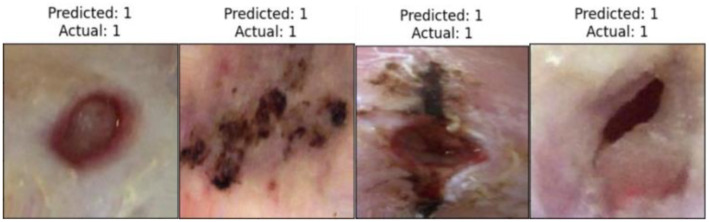
A sample of correctly classified infection images in the infection classification task performed by E-DFu-Net, showcasing its accuracy and effectiveness.

Figure 11A displays the ROC curves for CNN models with the proposed head model in ischemia classification. Conversely, Figure 11B shows the ROC curves for the same head model in infection classification. The curves indicate that the EfficientNetB0 CNN model, using the proposed head model, outperforms other models like DenseNet121, ResNet101, InceptionV3, InceptionResNetV2, VGG16, and MobileNetV2 in both tasks. Figure 12 displays the confusion matrix for E-DFu-Net in ischemia classification. Figure 13, on the other hand, shows the confusion matrix for E-DFu-Net in infection classification. In Figure 14, examples of ischemia accurately classified by E-DFu-Net can be easily identified. Meanwhile, Figure 15 presents images correctly classified as infections by E-DFu-Net. Table 9 presents a comparison between the proposed model and other studies.

**Table 9:** A comparison of the proposed model with the related work

**Study**	**Model**	**Class**	**Acc.**	**Prec.**	**Sens.**	**Sp.**	**F1 score**	**AUC**
Goyal et al., [11]	Ensemble CNN. with SVM classifier	Ischemia	0.903	0.918	0.886	0.921	0.902	0.904
		Infection	0.727	0.735	0.709	0.744	0.722	0.731
Al-Garaawi et al., [24]	GoogLNet CNN	Ischemia	0.92	0.94	0.93	0.90	0.93	0.97
		Infection	0.73	0.73	0.74	0.71	0.76	0.81
Proposed work	(EfficientNetB0 + Head model)	Ischemia	0.965	0.959	0.971	0.958	0.965	0.965
		Infection	0.919	0.951	0.883	0.954	0.916	0.919

## Discussion

This research aimed to categorize DFU images into two separate groups: one representing ischemia and the other representing infection. The primary goal was to prevent the misdiagnosis of DFUs, addressing the confusion between DFUs and malignancies. The challenge of distinguishing DFUs from skin cancers, especially in elderly patients, could potentially be solved through the application of computer vision technology [38]. The approach proposed in this study is expected to aid in the development of more advanced automatic telemedicine systems through which the identification of DFUs can be performed. These systems, in turn, are expected to assist in making early and efficient diagnoses, leading to timely treatment. Furthermore, complications arising from diabetic foot problems can also be prevented. The study found that the EfficientNetB0 pretrained model surpassed other such pretrained models, including ResNet101, DenseNet121, InceptionV3, VGG16, InceptionResNetV2, and MobileNetV2, in two tasks: classifying ischemia and infection. The study also emphasized that the head model, a unique technique introduced in the study, could largely prevent pretrained CNN models from overfitting, leading to a better learning curve and, consequently, better performance of the models.

The results of this study surpass previous research using pretrained CNN models for classifying DFU images. Goyal et al. [11] utilized three pretrained models, achieving 90% accuracy for ischemia classification and 73% for infection. Al-Garaawi et al. [24] used a pretrained GoogLeNet CNN model, obtaining 92% accuracy for ischemia and 73% for infected DFUs. Our study achieved notably higher accuracy, reaching 97% for ischemia and 92% for infected tissues. Integrating the suggested head model with CNN models boosts their accuracy, reliability, and ability to handle new data. Most CNN models show improved performance on all evaluation metrics when this head model is used. The EfficientNetB0 pretrained CNN model, combined with the proposed head model, achieves the highest ischemia classification results, with an accuracy of 0.965, precision of 0.959, sensitivity of 0.971, specificity of 0.958, *F1* score of 0.965, and an AUC of 0.965. For infection classification, it also achieves top results with an accuracy of 0.919, precision of 0.951, sensitivity of 0.883, specificity of 0.954, *F1* score of 0.916, and an AUC of 0.919. In real-time scenarios, balancing validation metrics and processing time, DenseNet121 and MobileNetV2 are practical lightweight models. DenseNet121 provides the best trade-off for developing mobile applications for DFU diagnosis, highlighting challenges in using EfficientNetB0-based models for real-time diagnosis.

## Conclusion

This paper presented an efficient deep fusion upsampled network (E-DFu-Net) in pretrained models using an efficient Fusion Network. The research involved comparing the performance of various CNN-pretrained models with the proposed model. In this study, pretrained models like VGG16, ResNet101, EfficientNetB0, DenseNet121, InceptionV3, InceptionResNetV2, and MobileNetV2 were integrated with the proposed model. Transfer learning techniques and hyperparameter tuning were applied to all CNN models used in this work. Experiments were conducted on the binary classification of ischemia and infection. For ischemia classification, the binary classes are ischemia and non-ischemia. For infection classification, the binary classes are infection and non-infection. The experiments show that the proposed E-DFu-Net model overcomes the overfitting issues observed in all pretrained models of CNN and gives better results compared to most of the pretrained models of CNN, thus making these models ready to be implemented in clinical environments.

Based on the research findings, EfficientNetB0, a pretrained CNN model, outperformed other pretrained CNN models, such as DenseNet121, ResNet101, InceptionV3, InceptionResNetV2, VGG16, EfficientNetB0, and MobileNetV2 for both ischemia and infection classification. Although the new proposed model was integrated and evaluated, according to the experimental results, EfficientNetB0 still produced the best performance in both ischemia and infection classification in this study with 97% accuracy in ischemia classification and 92% accuracy in infection classification. For future research, this work can be applied to a larger dataset and explore disease classification methodologies with improved image analysis techniques for disease classification.

DFUs can be seamlessly integrated into healthcare settings using simple tools that can be easily operated by clinical staff. The workflow is compatible with existing Electronic Health Record (EHR) systems and utilizes the Fast Healthcare Interoperability Resources (FHIR) format. This setup grants easy access to the diagnostic tool and data collection tool [R2] and its utilization can be readily adopted in clinical environments. The integration of the interaction of the user interface (UI) with the existing EHR systems was necessary to ensure the proper functioning and compatibility of our DFU diagnostic system within busy healthcare settings [39] such as: emergency rooms (ERs), wound care clinics, primary care offices and diabetes care centers.

## Data Availability

The used dataset is available by request at http://www2.docm.mmu.ac.uk/STAFF/M.Yap/dataset.php.
